# Implantable cardioverter defibrillators for primary prevention of death in left ventricular dysfunction with and without ischaemic heart disease: a meta-analysis of 8567 patients in the 11 trials

**DOI:** 10.1093/eurheartj/ehx028

**Published:** 2017-02-21

**Authors:** Matthew J. Shun-Shin, Sean L. Zheng, Graham D. Cole, James P. Howard, Zachary I. Whinnett, Darrel P. Francis

**Affiliations:** Imperial College London, National Heart and Lung Institute, Hammersmith Hospital Campus, B Block, 2nd floor, NHLI - Cardiovascular Science, Du Cane Road, W12 0NN London, UK

**Keywords:** Implantable cardiac defibrillators, Meta-analysis, Ischaemic heart disease, Cardiomyopathy, Non-ischaemic, Heart failure

## Abstract

**Aims:**

Primary prevention implantable cardioverter defibrillators (ICDs) are established therapy for reducing mortality in patients with left ventricular systolic dysfunction and ischaemic heart disease (IHD). However, their efficacy in patients without IHD has been controversial. We undertook a meta-analysis of the totality of the evidence.

**Methods and results:**

We systematically identified all RCTs comparing ICD vs. no ICD in primary prevention. Eligible RCTs were those that recruited patients with left ventricular dysfunction, reported all-cause mortality, and presented their results stratified by the presence of IHD (or recruited only those with or without). Our primary endpoint was all-cause mortality. We identified 11 studies enrolling 8567 participants with left ventricular dysfunction, including 3128 patients without IHD and 5439 patients with IHD. In patients without IHD, ICD therapy reduced mortality by 24% (HR 0.76, 95% CI 0.64 to 0.90, *P* = 0.001). In patients with IHD, ICD implantation (at a dedicated procedure), also reduced mortality by 24% (HR 0.76, 95% CI 0.60 to 0.96, *P* = 0.02).

**Conclusions:**

Until now, it has never been explicitly stated that the patients without IHD in COMPANION showed significant survival benefit from adding ICD therapy (to a background of CRT). Even before DANISH, meta-analysis of patients without ischaemic heart disease already showed reduced mortality. DANISH is consistent with these data. With a significant 24% mortality reduction in both aetiologies, it may no longer be necessary to distinguish between them when deciding on primary prevention ICD implantation.

## Introduction

Implantable cardiac defibrillators (ICD) are established as preventing death in patients with left ventricular dysfunction and ischaemic heart disease (IHD).^1^ In patients without IHD, however, ICDs are already considered controversial,^2^ and recent trial data have been interpreted as indicating that they are not beneficial.^3^

We set out to analyse the totality of RCT data of ICD vs. no ICD therapy in primary prevention of mortality in patients with left ventricular dysfunction.

## Methods

### Eligibility and search strategy

We identified all reports of studies of the use of ICD therapy against no ICD therapy for primary prevention in patients with left ventricular systolic dysfunction, in which outcome data was available stratified by the presence of IHD, or recruited only one of these two groups. We included cardiac resynchronization therapy (CRT) RCTs that included a defibrillator arm (CRT-D) and a cardiac resynchronization pacing only arm (CRT-P). We did not include comparisons between CRT-D and no device.

Pubmed (1st January 1946 to 18th December 2016), EMBASE (1st January 1974 to 18th December 2016), and the Cochrane Central register for randomized controlled trials using the search strategy detailed in Supplementary material online, Appendix S1. Only articles in English were considered. Reference lists and relevant systematic reviews were hand-searched for additional publications. No published protocol exists for this systematic review and meta-analysis.

### Data abstraction

Data was independently extracted by two authors (SZ, MJS), including year, participants, intervention, and outcomes. Disagreements were resolved by discussion with a third reviewer (DPF). The risk of bias was independently assessed by two authors (SZ, MJS). We sought data on the primary outcome measure of all-cause mortality. Secondary outcome measures included cardiovascular mortality and sudden cardiac death. We also collected data on specific ICD associated complications including inappropriate shocks and device-related infections. We abstracted reported hazard ratios with confidence intervals, and appropriately transformed them for meta-analysis. If hazard ratios or their confidence intervals were not available, but Kaplan-Meier plots were available, we extracted the underlying data using Digitizer^4^ and converted to hazard ratios and their standard errors.^5^ If a trial^6^ randomized patients to control, CRT-Defibrillator, and CRT-Pacemaker; and only presented data stratified by aetiology for the CRT-Defibrillator vs. control, and CRT-Pacemaker vs. control comparisons; the effect of the defibrillator component was determined by indirect comparison of the CRT-Defibrillator vs. the CRT-Pacemaker arms. The steps used to calculate the hazard ratio effect of the defibrillator component, and derive its confidence interval, for the groups with and without IHD separately, are shown in Supplementary material online, Appendix S2, and are based on formulae from Tierney *et al.*^7^

If hazard ratio data were unavailable^8^ we extracted risk ratios.

### Risk of bias assessment

We used the Cochrane Risk of Bias Tool^9^ to assess all trials for bias across six domains (selection, performance, detection, attrition, reporting, and other).

### Data analysis

Where appropriate, we quantitatively synthesised the extracted hazard ratios and risk ratios using a random-effects meta-analyses with the Restricted Maximum Likelihood (REML) estimator. We calculated the annualized mortality rate across for each aetiology by dividing the overall mortality rate in the control group by the mean follow-up time, and weighting by study size. The *I*^2^ statistic was used to measure heterogeneity of trial results.^10^ We carried out a sensitivity analysis for patients without IHD by omitting each of the trials in turn and repeating the meta-analysis. Publication bias was graphically assessed using Funnel plots, with Egger’s test for asymmetry.^11^ Data were analysed using “R”,^12^ and the package “metafor”.^13^ The PRISMA checklist is included as Supplementary Data.^14^

## Results

**Figure 1:**
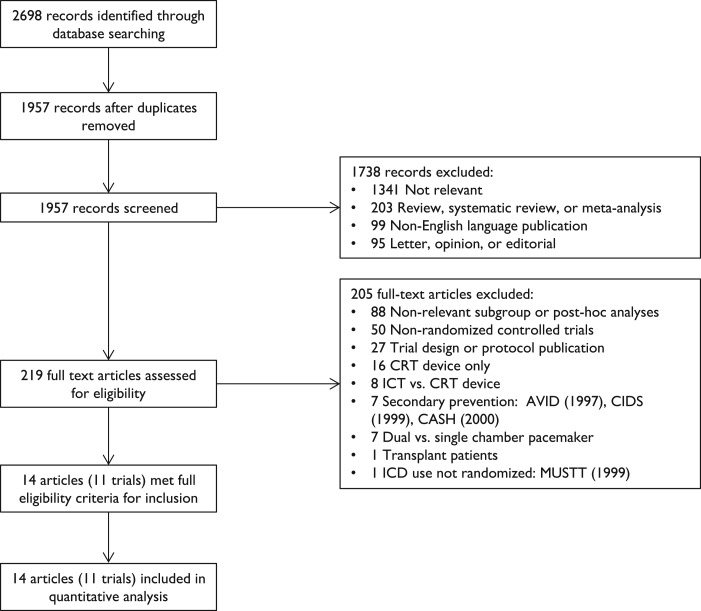
Study flow chart.

The primary search yielded 2698 records, which were processed as shown in the study flow chart (*Figure **1*). Full-text was independently reviewed for 219 articles and 11 trials of ICD therapy for primary prevention were included. Three additional articles reported secondary outcomes for included trials.^15–17^ Two trials enrolled patients with left ventricular dysfunction regardless of aetiology,^6^^,^^18^ four trials enrolled patients exclusively without IHD,^8^^,^^19–21^ three exclusively with chronic IHD,^22–24^ and two trials exclusively after an acute myocardial infarction.^25^^,^^26^ One trial^8^ used amiodarone as the comparator, all other trials continued prescribed therapy.

Three trials^27–29^ were excluded as they recruited patients resuscitated from an arrhythmic cardiac arrest, with an ICD inserted as secondary prevention. One trial^30^ was excluded as, whilst it was a randomized controlled trial, allocation to insertion of an ICD was not randomized.

A total of 8567 participants were enrolled (4371 ICD therapy, 4196 control), 3128 without IHD and 5439 with IHD (*Table **1*, study characteristics).

**Table 1:** Study characteristics

Trial	CABG-Patch	MADIT I	MADIT II	CAT	AMIOVIRT	DEFINITE	DINAMIT	COMPANION	SCD-HeFT	IRIS	DANISH
Year	1996	1996	2002	2002	2003	2004	2004	2004	2005	2009	2016
Author	Bigger	Moss	Moss	Bänsch	Strickberger	Kadish	Hohnloser	Bristow	Bardy	Steinbeck	Kober
Intervention	ICD	ICD	ICD	ICD	ICD	ICD	ICD	CRT-D	ICD	ICD	ICD
Control	SMT	SMT	SMT	SMT	Amiodarone	SMT	SMT	CRT-P	SMT	SMT	SMT
LVEF cut-off	<36%	≤35%	≤30%	≤30%	≤35%	<36%	≤35%	≤35%	≤35%	≤40%	≤35%
Randomized (N)	900	196	1232	104	103	458	674	1520	1676	898	1116
Without IHD	–	–	–	100% (*n* = 104)	100% (*n* = 103)	100% (*n* = 458)	–	44% (*n* = 669)	47% (*n* = 792)	–	100% (*n* = 1116)
With IHD	100% (*n* = 900)	100% (*n* = 196)	100% (*n* = 1232)	–	–	–	100% (*n* = 674)	56% (*n* = 851)	53% (*n* = 884)	100% (*n* = 898)	–
ICD group N	446	95	742	50	51	229	332	595	829	445	556
Follow-up (months)	32	27	20	66	24	29	30	15.8	45.5	37	67.6
Primary outcome	ACM	ACM	ACM	ACM	ACM	ACM	ACM	ACM and hospitalization	ACM	ACM	ACM
Inclusion criteria	Undergoing CABG, abnormal ECG	MI, NSVT, NYHA 1-3	MI, NYHA 1–3	Recent DCM diagnosis, NYHA 2-3	NYHA 1-3, asymptomatic	Symptomatic DCM, ambient arrhythmias	Recent MI	NYHA 3–4, recent HF hospitalization	NYHA class 2–3, OMT	Recent MI	NYHA 2-4, raised NT- proBNP
Exclusion criteria	Sustained VT or VF	Cardiac arrest, syncopal VT,	MI within 1month	Valvular, HCM or restrictive, prior MI	Syncope	NYHA 4, familial cardiomyopathy	NYHA 4			NYHA 4, ventricular arrhythmia before or ≥ 48 h after	
EP inclusion criteria	QRS ≥ 114 or other signal averaged ECG abnormalities	NSVT (3–30 beats at rate >120)	VE	Excluded VT, VF, symptomatic brady	Asymptomatic NSVT (>3 beats, HR > 100, lasting <30s)	NSVT (3-15 beats, HR < 120) or < 10 PVC/h	None	QRS≥120 ms, PR ≥ 150 ms, SR	None	HR ≥ 90, or NSVT (≥3 beats, HR ≥ 150)	None
IHD definition	Undergoing CABG	Q wave or cardiac enzyme positive MI	Q wave, cardiac enzymes, fixed defect nuclear scan, akinesis ventriculography, CAD on angio	No stenosis > 70% at coronary angiography	Absent CAD or out of proportion to CAD	Clinically significant CAD on angio or negative stress imaging	Recent MI	Not specified	≥75% narrowing of major artery, prior MI	STEMI or NSTEMI	No significant CAD on invasive or CT angiogram, or normal MPS. Allowed 2 stenosed coronaries if felt not significant.
Time after MI	–	>3 weeks	>1 month	NA	NA	NA	6–40 days	–	–	5–31 days	NA
ICD type	Epicardial	Epicardial 47%	Transvenous	Transvenous	Transvenous	Transvenous	Transvenous	Transvenous	Transvenous	Transvenous	Transvenous
		Transvenous 53%									
CRT implantation permitted	–	–	–	–	–	Yes	–	Yes	–	–	Yes
Age (mean±sd)	64±9	63	65 ± 10	52 ± 11	59 ± 12	58 (range 20–84)	62 ± 11	67	60	63 ± 11	64
Male	84%	92%	85%	80%	70%	71%	76%	68%	77%	77%	73%
ACEi/ARB	54%	62%	70%	96%	86%	97%	95%	89%	96%	82%	97%
BB	21%	23%	70%	4%	52%	85%	87%	68%	69%	98%	92%
CRT	0%	0%	NR	NR	NR	2%	NR	100%	NR	NR	58%
LVEF	27% (Mean)	26% (Mean)	23% (Mean)	24% (Mean)	23% (Mean)	21% (Mean)	28% (Mean)	21% (Median)	25% (Median)	35% (Mean)	25% (Median)
QRS width (ms)	NR (73% >100 ms)	NR	NR (51% > 120 ms)	108	NR	115	106	160	NR	NR	CRT 160No CRT 108
QRS normal	NR	NR	49%	64%	NR	NR	NR	NR	NR	NR	NR
QRS abnormal	LBBB 11%	LBBB 8%	LBBB 19%	LBBB 30%	LBBB 48%	LBBB 20%	NR	LBBB 71%	NR	LBBB 8%	CRT LBBB 94%, RBBB 3% No CRT LBBB 17%, RBBB 5%
			RBBB 8%	RBBB 1%	RBBB 12%	RBBB 3%		RBBB 11%			
NYHA I			37%	0%	16%	22%	13%	0%	Excluded	Recruited	0%
NYHA II	73% (II and III)	65% (II and III)	35%	65%	64%	57%	60%	0%	Recruited	Recruited	53%
NYHA III			24%	35%	20%	21%	27%	86%	Recruited	Recruited	45%
NYHA IV			4%	0%	0%	0%	0%	14%	Excluded	Excluded	1%
Hypertension	NR	42%	53%	NR	63%	NR	46%	NR	56%	66%	31%
Diabetes	38%	6% (IDDM)	36%	NR	34%	23%	30%	NR	31%	34%	19%
Atrial fibrillation	NR	NR	NR	NR	NR	25%	NR	NR	15%	14%	22%

ICD, implantable cardioverter defibrillator; CRT-D/P, cardiac resynchronization therapy-defibrillator/pacemaker; SMT, standard medical therapy; ACM, all-cause mortality; MI, myocardial infarction; NSVT, non-sustained ventricular tachycardia; NYHA, New York Heart Association Functional Classification; DCM, dilated cardiomyopathy; HF, heart failure; OMT, optimal medical therapy; VE, ventricular ectopics; VT, ventricular tachycardia; VF, ventricular fibrillation; CAD, coronary artery disease; MPS, myocardial perfusion scintigraphy; CABG, coronary artery bypass graft; NA, not applicable.

### Risk of bias assessment

Trial quality was assessed using Cochrane risk of bias tool (*Table **2*). There was no effective blinding of therapy in any of the trials. We assessed our primary end-point of all-cause mortality as having a low risk of bias. End-points requiring clinical judgement, such as sudden cardiac death and cardiovascular death, are at risk of bias if assessors are not blinded. Only five^6^^,^^8^^,^^20^^,^^21^^,^^26^ of the eleven trials reported on procedures to blind end-point assessment. Secondary outcomes were poorly reported, and often used different statistical measures to the primary outcome.

**Table 2:** Risk of bias

Trial	CABG-Patch	MADIT I	MADIT II	CAT	AMIOVIRT	DEFINITE	DINAMIT	COMPANION	SCD-HeFT	IRIS	DANISH
Year	1996	1996	2002	2002	2003	2004	2004	2004	2005	2009	2016
Author	Bigger	Moss	Moss	Bansch	Strickberger	Kadish	Hohnloser	Bristow	Brady	Steinbeck	Køber
Random sequence generation (selection bias)	Low risk	Unclear–not reported	Unclear–not reported	Low risk–central randomization	Unclear–not reported	Unclear-not reported	Low risk–central randomization with stratification	Unclear–not reported	Low risk	Low risk	Low risk–Web-based randomization with stratification
Allocation concealment (selection bias)	Unclear	Unclear–not reported	Unclear–not reported	Low risk– “closed envelopes with the assigned study group were sent to each centre … envelopes were opened when a patient was enrolled”	Unclear–not reported	Unclear-not reported	Unclear–not reported	Unclear–not reported	Low risk	Unclear–not reported	Low risk
Blinding of participants and personnel (performance bias)	High–“nature of the intervention precluded the blinding of investigators or patients”	High risk	High risk	High risk	High risk	High risk	High risk	High risk–“patients, physicians… were not blinded to the treatment assignments”	High risk	High risk	High risk
Blinding of outcome assessment (performance bias)	Unclear–“accumulating data were reviewed by an independent Data and Safety Monitoring Board”, but no report of whether outcomes were blindly assessed	Unclear–“two member end-point subcommittee reviewed information on the causes and circumstances of deaths”, but no report on whether blinded	Unclear–not reported	Unclear–not reported	Low–“events committee determined the cause of death” … “independently evalutated all information available” and “to assure a blinded review, all references to amiodarone or ICD therapy was removed from the reviewed documents”	Low - “cause of death was determined by an events committee… unaware of patients’ treatment assignment”	High – “ascertainment of the cause of death was the responsibility of the local investigators”, but a “blinded central validation committee independently reviewed information on all deaths”	Low–“steering committee and endpoint committee were unaware of the treatment assignments”	Unclear–not reported	Low–“adverse-event committee that was unaware of the treatment assignments classified” the causes of death	Low–“endpoint classification committee, the members of which were unaware of the treatment assignments, used prespecified criteria to adjudicate all prespecified cinical outcomes”
Incomplete outcome data (attrition bias)	Low risk	Low risk	Low risk	Low risk	Low risk	Low risk	Low risk	Low risk	Low risk	Low risk	Low risk
Selective reporting (reporting bias)	Low risk	Low risk	Low risk	Low risk	Low risk	Low risk	Low risk	Low risk	Low risk	Low risk	Low risk
Other bias	Trial funded by CPI/Guidant who supplied devices, but had no role in design, analysis, interpretation or writing.	Trial funded by CPI/Guidant who supplied devices, but had no role in design, analysis, interpretation or writing.	Trial funded by CPI/Guidant who supplied devices, but had no role in design, analysis, interpretation or writing.	Trial funded by CPI/Guidant who supplied devices, but had no role in design, analysis, interpretation or writing.	Supported in part by an unrestricted research grant from the Guidant Corporation	Trial funded by St Jude who supplied devices, but had no role in design, analysis, interpretation or writing.	Trial funded by St Jude who supplied devices, but had no role in design, analysis, interpretation or writing.	Trial funded by Guidant who supplied devices, but had no role in design, analysis, interpretation or writing.	Trial funded by Medtronic who supplied devices, but had no role in design, analysis, interpretation or writing.	Trial funded by Medtronic who supplied devices and had access to the final pre-submission manuscript	Trial funded by Medtronic, St Jude, TrygFonden, but had no role in design, analysis, interpretation or writing.

### Populations studied

Across the 11 trials, the mean age was 63.1 years. Most trials enrolled patients with an EF ≤ 35%; two trials enrolled those with an EF ≤ 30%,^19^^,^^23^ and one enrolled those with an LVEF ≤ 40%.^26^

All trials included patients with NYHA Class III symptoms. In 5 trials only patients who were NYHA Class II and III were included. Three trials included patients with NYHA Class IV symptoms, but these accounted for only a small proportion of patients (14%, 4%, 1%). One trial^6^ did not recruit NYHA Class II patients. 5 trials^8^^,^^21^^,^^23^^,^^25^^,^^26^ included NYHA Class I patients.

The electrophysiology inclusion criteria varied between the trials with 6 trials enrolling based on previous NSVT or ectopics, and 5 having no specific electrophysiological inclusion criteria.

In one trial,^22^ ICDs were placed with epicardial leads during coronary artery bypass grafting (CABG) surgery. In one trial,^24^ 47% were placed with epicardial leads and 53% placed with transvenous leads. In all other studies transvenous leads were used. The studies enrolling patients with chronic IHD recruited patients at least 3 weeks after previous MI; those enrolling patients with acute MI within 31 days^26^ or 40 days of an MI.^25^ Baseline characteristics, inclusion, and exclusion criteria are detailed in *Table **1*.

### Effect on all-cause mortality

#### Left ventricular dysfunction without ischaemic heart disease

**Figure 2:**
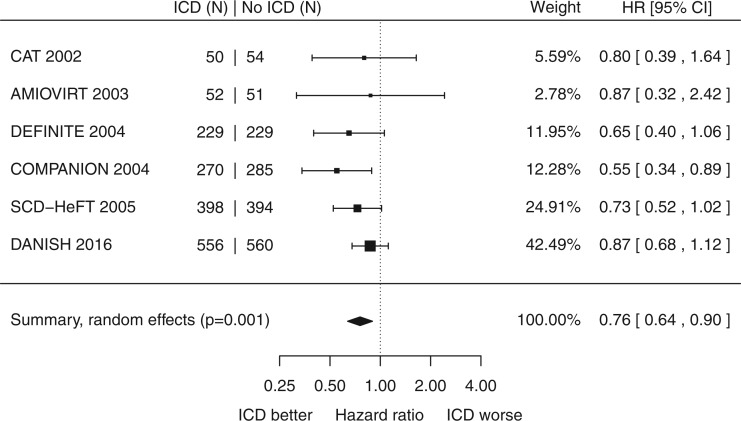
Title: Left ventricular dysfunction without ischaemic heart disease: impact of primary prevention ICD on all-cause mortality.

Across the 3128 patients without ischaemic heart disease, there was a significant reduction in all-cause mortality with minor heterogeneity (HR 0.76, 95% CI 0.64 to 0.90, *P* = 0.001, *I*^2 ^=^ ^3%, *Figure **2*). The annualized mortality rate in control patients was 5.4%.

A sensitivity analyses, carried out by omitting each of the trials in turn, in each case shows a statistically significant consensus reduction in mortality (see Supplementary material online, Appendix S4). A funnel plot did not show any significant asymmetry (Egger’s test *P* = 0.5, Supplementary material online, Appendix S5).

#### Left ventricular dysfunction with ischaemic heart disease

**Figure 3:**
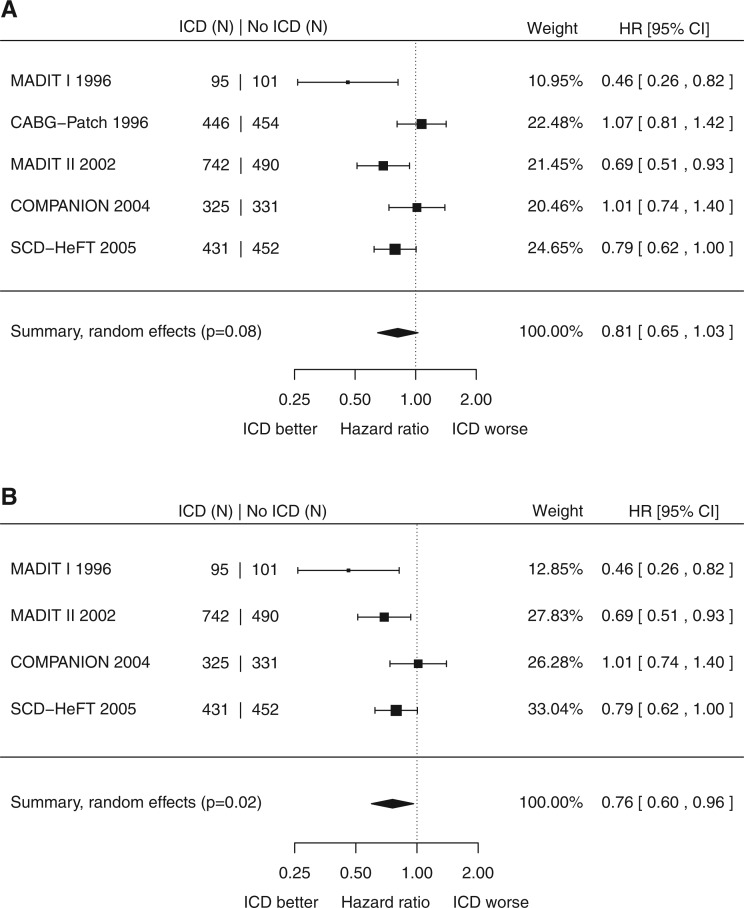
*(A)* Title: Left ventricular dysfunction with ischaemic heart disease: impact of primary prevention ICD on all-cause mortality. *(B)*. Title: Left ventricular dysfunction with ischaemic heart disease: impact of primary prevention ICD implanted during a dedicated procedure on all-cause mortality.

Across the 3867 patients in all trials of primary prevention ICD therapy with ischaemic heart disease and no recent MI, there was a non-significant reduction in all-cause mortality (pooled HR 0.81, 95% CI 0.65 to 1.03, *P* = 0.08, *Figure **3**A*). However, there was substantial heterogeneity (*I*^2 ^=^ ^62%). One trial^22^ was unique in inserting the ICD at the time of CABG surgery. There was a 16% higher infection rate in the ICD group, with 4.3% requiring removal. Current practice is to minimize infection risk by implanting the cardiac device separately from any open surgery. Running the analysis for the trials that tested this approach showed a significant reduction in mortality (HR 0.76, 95% CI 0.60 to 0.96, *P* = 0.02, *I*^2^ 52%, *Figure 3B*). The annualized mortality rate in the control patients was 11.3%. A funnel plot did not show any significant asymmetry (Egger’s test *P* = 0.2, Supplementary material online, Appendix S5).

#### Left ventricular dysfunction with acute myocardial infarction

**Figure 4:**
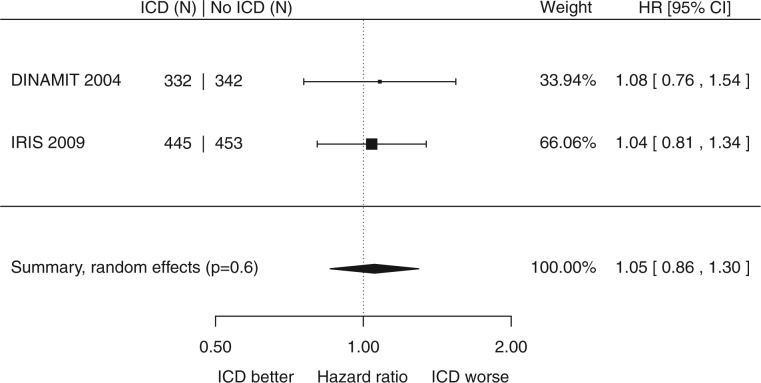
Left ventricular dysfunction with acute myocardial infarction: impact of primary prevention ICD on all-cause mortality.

In the 2 trials that enrolled 1572 patients after an acute MI, ICD therapy did not cause a significant reduction in mortality (HR 1.05, 95% CI 0.86 to 1.30, *P* = 0.6, *I*^2 ^=^ ^0%, *Figure 4*). The annualized event rate in the control patients was 7.6%. Supplementary material online, Appendix S5 contains the funnel plot.

#### Effect on secondary outcomes

Secondary outcomes were inconsistently reported with not all trials presenting data. Some data were presented as raw counts from which risk ratios could be derived, and some as hazard ratios. ICD therapy was consistently associated with a statistically significant reduction in hazard ratio and risk ratios for all three groups (without IHD, with IHD, and acute MI) for sudden cardiac death (without IHD HR 0.4 RR 0.29; with IHD HR 0.38 RR 0.41; acute MI HR 0.49, RR 0.57, Supplementary material online, Appendix S3).

## Discussion

Based on high-quality data from RCTs, this meta-analysis finds that primary prevention ICDs reduce all-cause mortality in patients with left ventricular dysfunction both with and without IHD. No benefit from ICDs is seen in the setting of acute myocardial infarction. These findings are consistent with the current ESC guideline recommended management.^31^^,^^32^

### Patients without ischaemic heart disease

There has been controversy over the utility of ICDs in patients without IHD. Many of the published guidelines make a distinction between the aetiologies with respect to the level of evidence on which their recommendations are made. The 2015 European Society of Cardiology (ESC) ventricular arrhythmia guidelines,^31^ and the 2016 ESC heart failure guideline^32^ give ICDs for primary prevention a 1A recommendation for an ischaemic aetiology, and 1B for a non-ischaemic aetiology. Indeed, this uncertainty was the stimulus for conducting the recent DANISH study. Subsequent commentary^3^ has added to the uncertainty.

Part of this uncertainty may have arisen as mortality rate in patients without IHD is lower than those with IHD (5.4%/year vs. 11.3%/year, respectively), and consequently the confidence intervals are wider for individual trials.

However, all the point estimates lie in the range 0.55 to 0.87, and the trials showed minimal heterogeneity (*I*^2 ^=^ ^3%). The group without IHD in COMPANION was, even on its own, statistically significant for a reduction of all-cause mortality with ICD (see Supplementary material online, Appendix S2), although this was not the chosen central message of the COMPANION primary publication.

Our meta-analysis confirms a statistically significant reduction in all-cause mortality by primary prevention ICD in patients without IHD. Whilst only one trial was individually significant, the point estimates from all 6 trials were in the same direction, suggestive of benefit. Furthermore, even omitting both COMPANION and the recent DANISH trial from the meta-analysis still produces a statistically significant consensus reduction in mortality (see Supplementary material online, Appendix S4).

### Patients with ischaemic heart disease

This meta-analysis supports the current consensus that ICDs reduce all-cause mortality in left ventricular dysfunction with IHD, in the trials that use the current clinical convention of a dedicated device implant procedure. Interestingly, the reduction in hazard ratio is numerically the same (24%) in patients with and without IHD. Consequently, when considering ICD therapy, distinctions between the two groups may be unnecessary.

In acute myocardial infarction, however, there is no indication of a reduction in all-cause mortality.

### Difference between this meta-analysis and previous meta-analyses

Our meta-analysis is the first to include the results of the patients without IHD from the COMPANION and DANISH trials. Other meta-analyses^33^ have omitted COMPANION, presumably because the paper did not display the hazard ratio explicitly. However, the hazard ratio and its confidence interval can be calculated from the steps shown in Supplementary material online, Appendix S2. The current meta-analysis therefore provides important new information regarding the role of ICD therapy in patients with left ventricular dysfunction without IHD.

### Study limitations

Any meta-analysis can only examine studies that have actually been carried out. Different studies took different approaches to recruitment. However, it is notable that all six non-ischaemic trial results were concordant not only in the direction of effect, but also the approximate magnitude, with the *I*^2^ statistical test showing minor heterogeneity.

In the case of the COMPANION trial, the hazard ratio was calculated using the information published in the primary publication by steps shown in Supplementary material online, Appendix S2. The original publication did not comment on this hazard ratio. It is wise to be cautious of results of sub-group analyses, because many such analyses are possible and some will be positive by chance alone. However, the single most important dichotomy in current guidelines^31^^,^^32^ for primary prevention ICDs in left ventricular systolic dysfunction is the presence vs. absence of ischaemic heart disease. Therefore, this sub-group analysis need not be assumed to be a random result selected from many possible sub-groups analyses. Moreover, all six groups of patients without ischaemic heart disease showed the same direction of effect. Furthermore, the finding is stable to the removal of any one trial (see Supplementary material online, Appendix S4).

Background medical therapy has improved over the time-course of these trials, with only 4% treated with beta-blockers in the CAT (2002), but 92% in DANISH (2016). Whilst the relative mortality-reduction effect size has remained remarkably consistent over time this will reduce the absolute effect size (when analysed over a fixed time window) of ICDs for primary prevention.

Our study could not consider the degree to which comorbidities might affect results. It has been noted that patients recruited into trials often have fewer comorbidities than those in the general population. The external validity of RCTs is always challenged by this, particularly in conditions such as heart failure where comorbidities may be frequent and severe.^34^ Furthermore, whilst this meta-analysis finds that stratifying by the presence or absence of ischaemic heart disease does not influence the mortality benefit of ICDs in primary prevention, other factors might. Supplementary material online, Appendix S4 includes data stratified by the presence or absence of CRT, but this analysis is hindered by the limited data in CRT group which is derived from COMPANION^6^ and a sub-group of DANISH.^20^ The 2013 ESC guidelines on cardiac pacing and cardiac resynchronization therapy^35^ similarly recognize that limited RCT data is available for the comparison between CRT-P and CRT-D. The guidelines suggest clinical conditions such as advanced or end-stage cardiac or renal disease may favour CRT-P over CRT-D.

### Clinical implications

The challenge facing clinical trials, as highlighted by McMurray,^3^ is that skilful modern treatment algorithms have reduced event rates down to low levels in the types of patients who would be eligible for, and willing to enter, randomized controlled trials; the annualized rate is 5.4% in patients without IHD. In light of this perhaps, we should pay maximal attention to information that RCTs give us.

The low event rate in the trials is why viewing multiple trials is necessary to see the survival benefit. However, the 24% risk reduction is as sizable as one might realistically hope for, for any intervention. This meta-analysis provides strong support for the role of primary prevention ICDs in patients with left ventricular dysfunction. A 24% risk reduction in all-cause mortality is comparable with other therapies which we recommend in heart-failure such as candesartan^36^ or an angiotensin-neprilysin inhibitor (HR 0.77, 0.84, respectively).^37^

## Conclusions

In patients with left ventricular dysfunction, primary prevention ICDs reduce mortality. ICDs reduce mortality by 24% in both patients with (*P* = 0.03) and without IHD (*P* = 0.0023).

When deciding on ICD therapy, classification of heart failure by aetiology may therefore not be useful.

## Supplementary material

Supplementary material is available at *European Heart Journal* online.

## Funding

This work was supported by the British Heart Foundation [grant numbers FS/14/27/30752 (MJSS), FS/12/12/29294 (GC), FS/13/44/30291 (ZW), FS/10/038 (DPF)].

**Conflict of interest**: M.J.S.S., S.Z., J.P.H., G.C., and D.P.F. declare no conflict of interest. ZW has received speaker fees from St. Jude, and a research grant unrelated to this work from Medtronic.

## Supplementary Material

Supplementary Data
