# Supplementary material for: Implantable cardioverter defibrillators for primary prevention of death in left ventricular dysfunction with and without ischaemic heart disease: a meta-analysis of 8567 patients in the 11 trials
Source: Eur Heart J. 2017 Feb 21;38(22):1738–46. doi: 10.1093/eurheartj/ehx028 (PMC5461475; doi:10.1093/eurheartj/ehx028)
Supplement: Supplementary Data [file ehx028_supp.zip › Appendix 1 - Search Strategy.docx]

Appendix 1

Pubmed (from 1^st^ January 1946)

1. *defibrillator* or *ICD*
2. *mortality* or *death*
3. Cochrane highly Sensitive Search Strategy for identifying randomized trials (<http://handbook.cochrane.org/chapter_6/box_6.4.d_cochrane_hsss_2008_sensprec_ovid.htm>)

EMBASE (from 1^st^ January 1974):

1. *defibrillator* or *ICD*
2. *heart failure* or *cardiomyopathy*
3. *mortality* or *death*
4. *randomi*ed controlled trial* or *randomi*ed trial*

Cochrane CENTRAL (from 1^st^ October 2016):

1. *defibrillator* or *ICD*
2. *heart failure* or *cardiomyopathy*
3. *mortality* or *death*

Searches run on 17^th^ December 2016

References of all included studies, and review/systematic review/meta-analysis articles identified in the search, were hand searched for additional references.

One additional article was identified through this method – Steinbeck et al. (IRIS), 2009
