# Supplementary material for: Implantable cardioverter defibrillators for primary prevention of death in left ventricular dysfunction with and without ischaemic heart disease: a meta-analysis of 8567 patients in the 11 trials
Source: Eur Heart J. 2017 Feb 21;38(22):1738–46. doi: 10.1093/eurheartj/ehx028 (PMC5461475; doi:10.1093/eurheartj/ehx028)
Supplement: Supplementary Data [file ehx028_supp.zip › Appendix 2 - COMPANION Calculation.docx]

Appendix 2

Calculated from data shown in:

Cardiac-resynchronization therapy with or without an implantable defibrillator in

advanced chronic heart failure. Bristow MR, Saxon LA, Boehmer J, Krueger S, Kass DA, De Marco T, Carson P, DiCarlo L, DeMets D, White BG, DeVries DW, Feldman AM; Comparison of Medical Therapy, Pacing, and Defibrillation in Heart Failure (COMPANION) Investigators. *N Engl J Med*. 2004;350(21):2140-50.

The hazard ratio comparing CRT-D vs CRT-P stratified by ischaemic versus non-ischaemic aetiology can be calculated from the provided data in the COMPANION trial. The figure below shows the calculation for the non-ischaemic group.

The paper provides the total number of patients in each group:

*N_a_ =* 127; *N_b_* = 285; *N_c_* = 270

The standard errors of the known log hazard ratios can be calculated from their confidence intervals


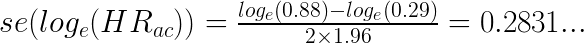


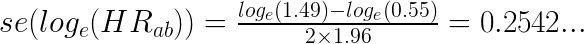


Then by using the following approximations and appropriate substitution *events_b_* and *events_c_* can be calculated


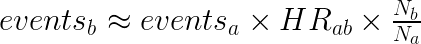


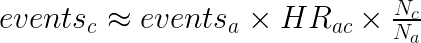


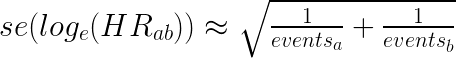


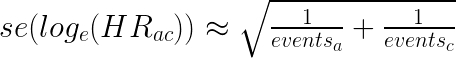


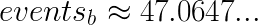


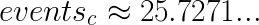


and then the standard error of *HR_bc_* can then be calculated


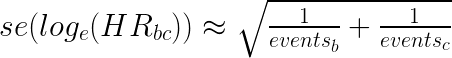


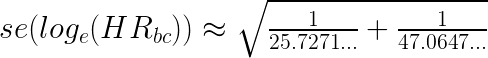


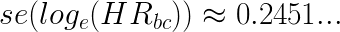


The lower (LCL) and upper (UCL) 95% confidence limits can then be calculated


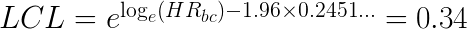


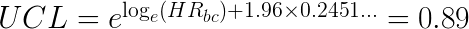


This calculation makes an assumption of exponential event rates, but proportional hazards methodology itself makes assumptions to arrive at a hazard ratio. Other estimates are possible but will be very close to the value shown above. Overall, the calculation presented here uses a conservative number of events, producing a conservative (i.e. slightly too wide) confidence interval.
